# In Vivo Imaging of Cobalt-Induced Ocular Toxicity in a Mouse Model

**DOI:** 10.3390/mps8010001

**Published:** 2025-01-02

**Authors:** Basel Obied, Galit Saar, Stephen Richard, Ygal Rotenstreich, Ifat Sher, Alon Zahavi, Nitza Goldenberg-Cohen

**Affiliations:** 1 The Krieger Eye Research Laboratory, Bruce and Ruth Faculty of Medicine, Technion—Institute of Technology, Haifa 3525433, Israel; basel.obied01@gmail.com (B.O.); steverit11@gmail.com (S.R.); 2 Magnetic Resonance Imaging Laboratory, Bruce and Ruth Faculty of Medicine, Technion—Institute of Technology, Haifa 3525433, Israel; sgalit@technion.ac.il; 3 Department of Ophthalmology, Sheba Medical Center, Tel Hashomer 52621, Israel; ygal.rotenstreich@sheba.health.gov.il (Y.R.); ifatsher@gmail.com (I.S.); 4 Faculty of Medicine, Tel Aviv University, Tel Aviv 6997801, Israel; alonzahavi@gmail.com; 5 Department of Ophthalmology and Laboratory of Eye Research, Felsenstein Medical Research Center, Rabin Medical Center, Petach Tikva 4941492, Israel; 6 Department of Ophthalmology, Bnai-Zion Medical Center, Haifa 3339419, Israel

**Keywords:** Anterion, optical coherence tomography, Spectralis and Anterion OCT, optic neuropathy, cobalt toxicity, mouse model, magnetic resonance imaging

## Abstract

Cobalt is a trace element, crucial for red blood cell formation and neurological function. Cobalt toxicity is often only diagnosed after severe manifestations, including visual impairment. We aimed to investigate whether optical coherence tomography (OCT) and magnetic resonance imaging (MRI) can effectively detect cobalt-induced ocular toxicity in a murine model. Five wild-type mice (WT, C57Bl6) received daily intraperitoneal cobalt chloride injections for 28 days with a dosage of 12.5 mg/kg. Another 5 WT mice served as controls. After 28 days, all mice underwent manganese contrast-enhanced MRI and OCT examinations. Macroscopic and histological analysis of the enucleated eyes were performed. MRI revealed an increased signal in the optic nerves of injected mice. Anterion OCT provided in vivo visualization of the entire eye, demonstrating incipient cataract formation in the cobalt-injected mice. Both Spectralis domain OCT and Anterion, followed by histological analyses, confirmed preserved retinal structure with decreased thickness in the cobalt-injected group, with only minor neuronal damage and cell loss. Optic nerve analysis demonstrated myelin loss and increased inflammation with high levels of reactive gliosis. This study demonstrates optic neuropathy induced by cobalt toxicity, as shown by increased optic nerve signal on MRI without significant retinopathy. Anterion OCT showed incipient cataracts in the anterior segment.

## 1. Introduction

Systemic cobalt toxicity is often difficult to detect. Environmental exposure to batteries, metal alloys, reagents, dyes, and beer additives has been implicated in systemic cobalt toxicity [1]. Medical implants and hip arthroplasties in particular have been associated with cobalt toxicity, as failed ceramic femoral head prostheses release cobalt and chromium metal ions into the systemic circulation [2]. Cobalt toxicity affects multiple organ systems, manifesting through a range of clinical symptoms, mainly congestive heart failure and cardiomyopathy [3,4,5,6]. It is associated with neurotoxic effects that manifest as cognitive impairments, peripheral neuropathy, and motor dysfunction [7,8,9]. Endocrinological disturbances, including hypothyroidism and adrenal insufficiency, have also been observed in affected individuals [1]. Clinical evidence associates cobalt toxicity with visual dysfunction [8,10,11,12,13,14,15,16]. Recent reports have highlighted that cobalt exposure can lead to damage of both the retina and the optic nerve, resulting in a spectrum of visual disturbances ranging from decreased visual acuity to more severe visual impairments.

Optical coherence tomography (OCT) and magnetic resonance imaging (MRI) are imaging techniques used in vivo to assess the structural pathways of the visual system in humans [17,18,19,20]. OCT delivers high-resolution, cross-sectional views of the retina and optic nerve, allowing clinicians to detect structural changes that may contribute to visual impairment [17]. MRI provides a broader perspective by imaging the visual pathways between the eye and the brain [21]. In this study, we utilized OCT and MRI to image in vivo murine eyes following cobalt-induced toxicity in order to assess both retina and optic nerve changes on imaging.

This study represents the pioneering demonstration of the entire mouse globe using Anterion OCT, achieving high-resolution imaging. We complemented Anterion OCT imaging with the traditional Spectralis OCT system for detailed retinal imaging.

Cobalt toxicity has been implicated in visual dysfunction. Here, we investigate the feasibility of in vivo imaging by two OCT methods and manganese-enhanced MRI to detect possible ocular cobalt toxicity. Subsequently, retinal tissues and optic nerves were extracted for histological and immunohistochemical examination.

## 2. Materials and Methods

**Mice and Experimental Design**

Cobalt chloride (CoCl_2_; Sigma Aldrich, St. Louis, MO, USA) was administered to 5 wild-type mice (WT, C57Bl6) by repeated daily intraperitoneal (IP) low-dose injection for 28 days. Five WT non-injected mice served as a control.

CoCl_2_ was dissolved in 100 µL saline solution, and the injection dosage was calculated per kilogram mouse body weight based on the literature [22]. Thus, in a 28-gr (±1.5 gr) mouse, the dose was 3.75 mg/mL, in 0.1 mL, or in other words, 12.5 mg/kg/day.

For IP injection, the mouse was tilted with its head slightly toward the ground. The 27-needle was inserted bevel-up into the lower right quadrant of the abdomen toward the head at a 30–40 angle to the horizontal plane. The plunger was pulled back to ensure negative pressure and 0.1 mL CoCl_2_ solution was injected at the predetermined concentration. Mice were euthanized by carbon dioxide asphyxiation after 28 days.

**Optical Coherence Tomography (OCT)**

Following ketamine/xylazine anesthesia, mice were placed on chinrests with the eye perpendicular to the machine. Eyes were locally anesthetized with 0.4% oxybuprocaine hydrochloride solution (Localin; Fishcer Pharmaceutical Labs Ltd., Tel Aviv, Israel) and pupils were dilated using a 0.5% tropicamide solution (Midramid; Fischer Pharmaceutical Labs Ltd., Tel Aviv, Israel) A powerless contact lens was placed on the eye. Acquisition was performed using anterior protocol. All scans were performed using Anterion OCT (Heidelberg Engineering GmbH, Heidelberg, Germany) and Heidelberg Spectralis (Heidelberg Engineering, Heidelberg, Germany). For the Anterion OCT, the following settings were used: Axial resolution: 10 microns, length of B-scans: 16.5 mm, breadth of B-scans: 14 mm, scan rate: 50,000 Hz, laser wavelength: 1300 nm. For the Heidelberg Spectralis, acquisition was performed using the anterior segment protocol.

**Manganese-Enhanced MRI (MEMRI)**

For manganese (Mn)-enhanced MRI (MEMRI), 2 µL of 50 mM isotonic manganese chloride (MnCl_2_) solution (Sigma-Aldrich, St. Louis, MO, USA) was injected intravitreally into both eyes. MRI scanning was performed 16–20 h later.

Images were obtained using a 9.4 T horizontal MRI system (Bruker Biospec, Ettlingen, Germany), equipped with an 86 mm inner diameter cylindrical volume coil for radiofrequency transmission and a 20 mm diameter surface coil for signal detection. We ensured imaging accuracy and consistency by regularly using phantom tubes provided by Bruker (Bruker Corporation, MA, USA) as part of our standard quality control procedures to monitor signal and image quality.

Animals were anesthetized with 1–2% isoflurane in oxygen (0.7 L/min) during imaging. Respiration was tracked using a monitoring system from Small Animal Instruments (Stony Brook, NY, USA), and body temperature was regulated with a heated water circulation system.

Mn-enhanced MRI imaging was conducted with a 3D T_1_ weighted fast low-angle shot (FLASH) sequence. The parameters used were TR/TE 15/4.7 ms and pulse 15^0^, field of view 19.2 × 19.2 × 15.5 mm^3^, in-plane resolution, 100 µm, matrix size 192 × 192 × 31, number of averages 4, at a total duration of 6 min.

For both the low-dose IP and control experimental groups, mice underwent MEMRI scanning procedures (n = 5 each). Subsequently, the average signal intensity was computed for both optic nerves of every individual mouse.

**Histology**

After 28 days, the eyes were removed, fixed in 4% formaldehyde, and incubated overnight at 4 °C in 30% sucrose prepared in phosphate-buffered saline (PBS, 1X; Biological Industries, Beit HaEmek, Israel). The tissues were then embedded in an optimal cutting temperature (OCT) compound (Scigen Scientific, Gardena, CA, USA). Cryosections, 10 µm thick, of both the globes and optic nerves were prepared, mounted onto slides, and stained with hematoxylin and eosin (H&E) for analysis using light microscopy (Fluoview X, Olympus, Tokyo, Japan). Each slide contained three sequential sections.

**Immunohistochemistry**

Retinal sections were mounted onto slides and rinsed with PBS before being blocked for 1 h in a solution containing 5% fetal calf serum and 1% Triton X-100. The sections were then incubated overnight at 4 °C with primary antibodies, including anti-IBA-1 (1:500, Abcam, Cambridge, UK), anti-Neun (1:500, Merck Millipore, Burlington, MA, USA), and anti-GFAP (1:500, Abcam, Cambridge, UK). Following PBS washes, the slides were treated with secondary antibodies for 1 h at room temperature: goat anti-rabbit Alexa Fluor 647 (1:1000, Abcam, Cambridge, UK) and goat anti-chicken IgG NL-577 (1:200, R&D Systems-Biotest, Minneapolis, MN, USA). DAPI (4′,6-diamidino-2-phenylindole, Molecular Probes Invitrogen, Eugene, OR, USA) was used to stain cell nuclei. Fluorescent images were captured using a Zeiss LSM700 confocal microscope (Munich, Germany).

**TUNEL Assays**

Sections from eyes embedded in optimal cutting temperature (OCT) compound were also evaluated for apoptosis. Longitudinal sections, 10 µm thick, were prepared and subjected to TdT-mediated dUTP nick-end labeling (TUNEL) assays using the fluorescein-labeled apoptosis detection kit (Roche Diagnostics GmbH, Mannheim, Germany). Fluorescent staining was analyzed with a confocal microscope (Zeiss LSM700), with detailed examination of each retinal layer. In the group receiving intraperitoneal (IP) injections, results were compared between injected and non-injected control mice.

**Data Analysis**

Medical image processing, analysis, and visualization (MIPAV) software (NIH; http://mipav.cit.nih.gov, accessed on 22 December 2022) was used for data processing. For manual segmentation of the optic nerve and eye structure and signal intensities measurements, Fiji ImageJ software (1.54f, National Institute of Health, Bethesda, MD, USA) and Python software (3.12) was used.

GFAP immunofluorescence intensity was measured using ImageJ software (version 1.54f, National Institutes of Health, USA). Consistent laser settings and sampling areas were maintained across all analyses. The inferior retina was excluded from the analysis due to minimal GFAP changes in this region. Rectangular regions of interest were defined, spanning the apical surface of the ganglion cell layer (GCL) to the basal surface of the outer nuclear layer (ONL). The mean fluorescence intensity for each retina was calculated from these defined areas [17].

**Statistical Analysis**

Statistical analyses were performed using GraphPad Prism (v.10.2.0, GraphPad Software, San Diego, CA, USA). For GFAP, TUNEL, and Neun analysis, the non-parametric Mann–Whitney U test was used. For OCT analyses and MEMRI average signal intensity calculations, an unpaired Student’s *t*-test was applied. A significance level of *p* < 0.05 was set for all tests.

To ensure the validity of our statistical inferences, we took several steps to validate the assumptions prior to conducting the statistical analyses. First, we assessed the normality of the data using the Shapiro–Wilk test. In instances where the data significantly deviated from normality, a non-parametric Mann–Whitney U test was employed to ensure robustness in our results.

## 3. Results

### 3.1. MRI

**Figure 1:**
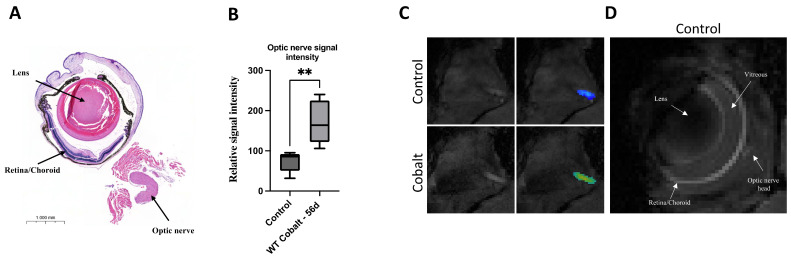
MEMRI showed signal enhancement of the optic nerve in the cobalt-treated group compared to controls. Illustrative histology section of a mouse eye (**A**). Represented T1-weighted MEMRI images of cross-section of the optic nerve showing signal enhancement of the optic nerve in the low-dose cobalt-treated group compared to controls. Five mice for each group (n = 5, each) (**B**,**C**). Representative field of view of MEMRI that illustrates the eye of a control mouse and structures including the lens, vitreous, retina, choroid, and optic nerve head (**D**). Statistics were performed using an unpaired Student’s test, with statistical significance indicated with asterisks: ** = *p* < 0.01.

Injection of Mn 16 h prior to MRI was used to assess the transport and accumulation of Mn in the optic nerves. This approach revealed abnormal Mn transport in the optic nerves of cobalt-treated mice compared to controls. Figure 1A,B show horizontal MRI sections of a mouse eye bulb after IP administration of cobalt daily for 28 days. Note the increased signal intensity of the optic nerves in the cobalt-treated mice compared to the control. MEMRI exhibited four distinct structures with different signal intensities including the lens, vitreous, retina, choroid, and optic nerve head (Figure 1C). MEMRI imaging could not identify any changes in the retina in the control and cobalt groups. Image intensity was used to detect Mn^+2^ accumulation in the optic nerves. Different degrees of Mn^+2^ enhancement indicated different levels of its accumulation across the nerves. The Mn intensity measured in the optic nerves of the cobalt-induced model were higher than those in the control group (Figure 1A).

### 3.2. OCT Imaging

**Figure 2:**
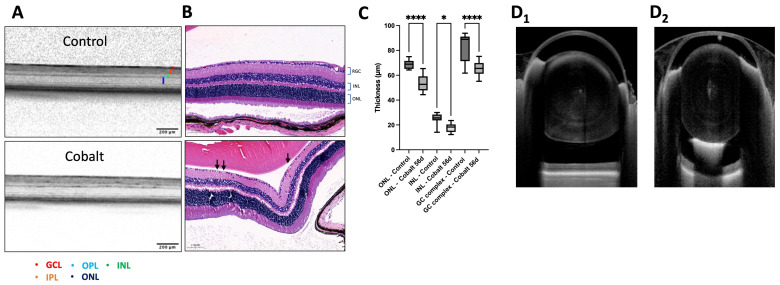
Retinal OCT of the control and cobalt group. Spectralis OCT showed retinal thickness measurement decrease in the cobalt-treated group. There was a decreased measurable thinning in the INL, ONL, and GC complex layers (black arrows show RGC loss) after 28 days of chronic low-dose cobalt administration (**A**–**C**). Five mice for each group (n = 5, each). Anterion OCT shows details of the anterior and posterior segment of the eye (**D_1_**). Anterion anterior segment OCT could identify lenticular hyperechoic areas, probably representing early-stage cataracts (**D_2_**). GCL: ganglion cell layer, IPL: inner plexiform layer, INL: inner nuclear layer, ONL: outer nuclear layer. Statistics were performed using an unpaired Student’s test with statistical significance indicated with asterisks: * = *p* < 0.05, **** = *p* < 0.0001.

OCT images were used to evaluate in vivo retinal layer anatomy and possible thinning due to chronic cobalt toxicity. Thinning was observed in the inner nuclear (INL) and outer nuclear (ONL) layers after 28 days of daily cobalt administration, indicating photoreceptor degeneration (Figure 2A,B). In addition, the ganglion cell layer (GCL) complex (GCL and inner plexiform layer) thickness was decreased in the cobalt group compared to the control group (Figure 2A,B).

The Anterion OCT demonstrated a section through the entire mouse globe (Figure 2C). The relative lens size was calculated in the mouse eye as 60% of its volume (Figure 2C). Anterior segment imaging showed mild lenticular opacities only in the cobalt-treated mice (Figure 2D_1_,D_2_).

Retinal images were captured from the identical region within the central retina. The Anterion OCT resolution showed preserved retinal integrity without being able to reveal any notable thickness changes in various layers.

### 3.3. Histology and Immunohistochemistry

**Figure 3:**
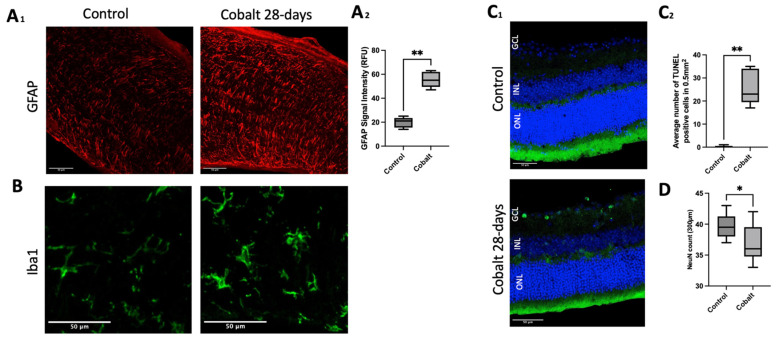
GFAP immunostaining for gliosis. Longitudinal sections of optic nerve of control and cobalt-treated mice were stained for astrocytes with GFAP (red). Increased GFAP expressing reactive astrocytes are observed in the cobalt-treated group (**A_1_**,**A_2_**). Iba1 immunostaining for microglia. Longitudinal sections of optic nerve were stained for microglia with iba1 (green). Microglia showed increased activation morphology (bigger soma and shorter branches) in the cobalt-treated group compared to control (**B**). TUNEL staining for retinal tissue. Representative micrograph of retina sections were evaluated for apoptosis by TUNEL assay. TUNEL positive cells were identified with green fluorescence and the nuclei of photoreceptors were counterstained with DAPI (blue). In the control group, only sparse to no TUNEL-positive cells were found, while in the cobalt-treated group, TUNEL-positive labeling was apparent in the INL and ganglion cell layer (**C_1_**,**C_2_**). NeuN immunostaining demonstrated decreased in NeuN positive cells in the cobalt-treated group compared to control (**D**). Outer nuclear layer (ONL); inner nuclear layer (INL); ganglion cell layer (GCL). Statistics were performed using a Mann–Whitney U test with statistical significance indicated with asterisks: * = *p* < 0.05, ** = *p* < 0.01. Scale bar: 50 microns.

In the control group, histological analysis confirmed the normal structure and thickness of the retina. In contrast, in the cobalt-treated group, histology revealed retinal thinning [INL (26.75 ± 4.73 vs. 20.01 ± 4.68; *p* < 0.0001), ONL (69.94 ± 4.46 vs. 54.54 ± 7.88; *p* < 0.01) GCL complex (81.35 ± 12.08 vs. 64.72 ± 6.91; *p* < 0.0001)]. Immunofluorescence analysis (IF) using TUNEL and Neun immunostaining further revealed significant neuronal cell loss in this group, as illustrated in Figure 3. GFAP immunostaining showed increased signal intensity in the optic nerve, while Iba-1 staining demonstrated microglial activation in the optic nerve (Figure 3A_1_,A_2_,B).

## 4. Discussion

In vivo imaging plays a crucial role in the assessment of ocular disease, yet has not been previously used for demonstrating retinopathy or neuropathy in cases of cobalt toxicity. In this study, we utilized a murine model to evaluate the feasibility of Spectralis OCT, Anterion OCT, and contrast-enhanced MRI in detecting subtle ocular changes induced by cobalt toxicity. MRI effectively showed reduced intracellular axonal flow of Mn following cobalt administration, while OCT imaging detected subtle structural changes in the lens and retina.

OCT, a non-invasive in vivo imaging technique, provided high-resolution ocular scans. It effectively visualized the proportions of the lens and globe, detected early lens opacities, and detailed anatomical landmarks. OCT is commonly used to provide in vivo information on retinal structure and disease, with resolution that can be equivalent to that of excisional biopsy and histopathology [17]. It is a non-invasive imaging technique that utilizes low-coherence light to capture detailed, cross-sectional images of biological tissues [23]. It has evolved into a reliable marker of disease advancement in both humans [24] and rodents [25,26]. High-resolution OCT systems can achieve axial resolutions in range of micrometers, typically ranging from 1 to 10 µm [19]. This high-resolution property allows for the identification of different layers of the retina, from the innermost retinal nerve fiber layer (RNFL) to the retinal pigment epithelium (RPE) and the choroid.

The demonstration of mouse retina with OCT requires adaptation of the machine or the use of specific OCT for mice. The need for high resolution OCT to detect subtle changes in the anterior or posterior segment are paramount in clinical settings [23]. Moreover, being non-invasive, OCT allows for the imaging of the same mouse for longitudinal follow-up over time. The limited use of this potent tool in animal research stemmed from the poor-quality images obtained in commercially available first- and second-generation time OCT devices when used in rodents [27], and only specifically adapted devices for the animal visual system improved imaging [26,28,29,30].

In our study, we utilized two OCT imaging modalities: Anterion OCT and Spectralis OCT. The Anterion OCT device used in this study is an OCT imaging system that combines advanced swept source-OCT technology with a range of diagnostic capabilities. It is designed for comprehensive ocular examinations, from anterior segment imaging to detailed assessments of the retina and optic nerve. Its high resolution, fast scan speed, and versatile applications make it a valuable tool for both clinical practice and research. While the resolution of the Anterion OCT device for retinal layers is relatively modest, its capability to visualize the entire mouse globe in detail was remarkable. The Spectralis OCT, on the other hand, provides a detailed and accurate imaging of the posterior ocular segment. Together, these OCT modalities enhance our ability to assess ocular health and disease. To the best of our knowledge, this study provides the first report of Anterion OCT usage in mice. It offered high-resolution visualization of the anterior segment of the mouse eye, including detailed views of the cornea and lens, as well as comprehensive imaging of the entire globe.

No discernible differences were observed between the cobalt-induced mouse model and control eyes, apart from the presence of mild anterior cataracts. The OCT imaging acquisition did not require any processing nor contrast injection, was easily performed, and clearly demonstrated the cornea, lens, and vitreous. The comprehensive analysis of overall ocular anatomy in vivo by the Anterion OCT could supplement histological studies. The Spectralis OCT demonstrated detailed retinal structures and layers, allowing for the measurement of retinal thickness. However, it failed to demonstrate destruction of the retinal layers in the cobalt-injected mice.

While OCT provided detailed globe imaging, MEMRI provided detailed optic nerve assessment. Enhancement of the optic nerve is in line with pathological imaging in human scans [31]. The high resolution of the 9.4 Tesla MRI system further enhanced its ability to discern subtle changes. In contrast to human studies where manganese (Mn) cannot serve as a contrast agent, in mice, Mn was injected intraocularly as an intracellular contrast agent. This approach relies on the normal functioning of retinal cells, particularly retinal ganglion cells involved in Mn transport across cell membranes for effective contrast enhancement [20,32,33]. Over the past two decades, MEMRI has emerged among advanced MRI techniques as a valuable tool for visualizing the structure and function of both the brain and peripheral structures. Mn ions exhibit paramagnetic features, rendering it a suitable contrast agent that effectively enhances T1-wieghted MRI intensities within local tissues due to the uptake of contrast over a specified duration [20,32,34].

In our study, we demonstrated that cobalt toxicity triggers a marked inflammatory response in the optic nerve, characterized by gliosis. This was evidenced by morphological changes in Iba-1 staining, a marker of microglial activation, which showed increased microglial activity, as well as elevated GFAP staining, a marker of astrocyte activation, indicating significant astrogliosis in the cobalt-treated group. These pathological alterations likely disrupt the normal functioning of the optic nerve, including the mechanisms responsible for Mn transport. As a result, the inflammation and gliosis impair Mn transport, leading to a reduction in signal intensity on MEMRI. This decrease in signal intensity reflects both compromised Mn transport and a diminished contrast enhancement in the optic nerve.

Additionally, our study observed the development of mild anterior cataracts in the cobalt-treated group, a finding that has not been reported in the literature in the context of cobalt toxicity. While the cataracts were identified using Anterion OCT, which allowed for detailed imaging of the anterior segment, we acknowledge that this observation has not been confirmed through alternative diagnostic methods. Cataract formation can result from metabolic changes in the lens, such as those observed in conditions like diabetes or kidney failure, and these changes are sometimes reversible. Given that cobalt-induced cataracts in humans have not been previously documented, this observation is novel and could be attributed to the first use of Anterion OCT imaging in a mouse model.

Although we interpret the cataract findings as indicative of a real pathological effect, it is important to recognize that they could represent reversible changes, potential imaging artifacts, or limitations inherent in the resolution of OCT. Further validation using complementary techniques, such as histological staining or PIXE analysis, would be valuable in confirming these observations. PIXE analysis of the lens material in future studies would provide additional insights into the underlying mechanisms of cobalt-induced cataract formation.

In this study, in vivo MEMRI was applied to assess retinal and ON degeneration in a cobalt toxicity mouse model. Using Mn^+2^ as an intracellular contrast agent, we quantified the signal intensity of the ON using MEMRI in both control and cobalt-treated mice. Signal intensity of the ON in the cobalt-treated group was increased compared to control. Axonal transport and accumulation of Mn^+2^ is an energetic process and depends on energy that is supplied by the mitochondria [35]. Cobalt toxicity can interrupt the citric acid cycle and the generation of ATP by aerobic cellular respiration and by that inhibiting the activity of respiratory chain enzymes and ATP production in mitochondria [36,37]. The resultant inhibition of mitochondrial function causes the disruption of ion hemostasis, energy and axonal transport deficit which could play a role in axonal demyelination and degeneration [38]. Three retinal layers were detected by the MRI and were categorized as nerve fiber layer/ganglion cell layer/inner plexiform layer, inner nuclear layer/outer plexiform layer, and photoreceptor cell outer nuclear layer/inner segment/outer segment. However, the 30 μm resolution employed MRI was not sufficient to detect retinal layer variation between the cobalt-treated and the control group.

Cobalt toxicity is known to cause reversible and even permanent visual dysfunction [2,15,16,39]. Delayed diagnosis and treatment of cobalt toxicity due to its low probability and the ambiguity of its signs and symptoms leads to irreversible visual damage in most cases [16]. Recently, there has been a significant increase in the reported cases of cobalt toxicity, especially in patients with hip metallic implants [2,12,15,39]. Therefore, early diagnosis is required to prevent and even reverse visual dysfunction. As the gross structures are not affected, high-resolution OCT is required to identify any retinal ganglion cell loss or photoreceptor degeneration.

In this study, the use of OCT marginally contributed to diagnosis. Although the Anterion OCT device provided views in relatively high resolution, MEMRI played a more significant role in assessing the visual pathway. Histological studies indicated that the optic nerve’s distinct structural and biochemical properties may contribute to its sensitivity to cobalt toxicity [40]. The anterior and posterior segments of the eye were delineated with high resolution, revealing subtle structural changes that could be overlooked in routine slit lamp and fundus examinations.

The mouse model is widely recognized as one of the best systems for studying optic nerve diseases, including ischemic neuropathy, due to its strong anatomical and physiological similarities to humans [41]. These models have been extensively used to investigate the mechanisms of nerve damage and recovery, providing valuable insights into human disease. Although direct extrapolation from mice to humans has limitations, previous studies have demonstrated that mouse models of cobalt toxicity can closely mimic human conditions, particularly in cases of hip implant-related toxicity, which involves both cardiac and neurotoxicity [42,43]. Our prior research has also shown that cobalt exposure in mice induces retinopathy and optic neuropathy, with electroretinogram (ERG) and retinal histology results that align with aspects of human pathology, further supporting the model’s relevance [44].

In the current study, we focused on enhancing imaging techniques to better visualize cobalt-induced ocular damage. While we acknowledge key anatomical differences between murine and human eyes—such as the mouse eye being approximately 1/5th to 1/6th the size of the human eye—Anterion OCT enabled us to capture the full eye globe in mice, making it useful for studying conditions like cataract formation. However, the resolution limitations of OCT in mice were evident. The Anterion OCT, though effective for non-invasive imaging from cornea to sclera, exhibited moderate resolution for measuring retinal thickness, which can limit its sensitivity for detecting subtle changes in retinal layers, such as those induced by cobalt toxicity. While Spectralis OCT was used to complement in vivo imaging by measuring retinal thinning in microns, the Anterion OCT was less sensitive for fine structural changes. Furthermore, TUNEL staining confirmed apoptosis across all retinal layers, underscoring the importance of histological analysis for definitive findings.

Despite the utility of OCT for in vivo imaging, the relatively low resolution in mice means that conclusions based on OCT imaging regarding fine structural changes, like retinal thinning or neuronal cell loss, should be interpreted with caution. This suggests the need for enhanced imaging techniques or alternative approaches to more comprehensively evaluate the ocular effects of cobalt toxicity.

Future research should focus on advancing OCT technology, improving its resolution and specificity for retinal layers, which would facilitate earlier diagnosis and better monitoring of visual dysfunction. Additionally, combining OCT with other complementary imaging techniques could offer a more complete assessment of both structural and functional changes in the visual pathway. Longitudinal studies examining the progression of cobalt-induced ocular damage and responses to treatment will be essential to develop more effective clinical management strategies and improve patient outcomes.

In summary, the combination of multimodal in vivo imaging, such as OCT and MRI, may allow for structural–functional comparison in a mouse model. It may provide sufficient information over time and enable exploration of the damage to the visual pathway. High-resolution OCT may increase the specificity of the findings and enable monitoring of the response to treatment.

## 5. Conclusions

In conclusion, in vivo studies may contribute to diagnosis of cobalt toxicity. MRI is widely used in assessing optic neuropathies, but in mice the use of Mn as intracellular contrast agent may serve as a functional test. MRI scans showed that optic neuropathy is more common than retinal dysfunction. The Anterion OCT provided high-resolution imaging of the entire mouse globe and the lens in particular. While acknowledging the limitations of current imaging modalities, particularly in detecting subtle changes in retinal morphology, our findings highlight the importance of continued research efforts in this area.

## Data Availability

The data that support the findings of this study are available from the corresponding author upon reasonable request.
